# Pregnancy outcomes in perinatally HIV-infected young women in Madrid, Spain: 2000-2015

**DOI:** 10.1371/journal.pone.0183558

**Published:** 2017-08-25

**Authors:** Luis M. Prieto, Carolina Fernández McPhee, Patricia Rojas, Diana Mazariegos, Eloy Muñoz, Maria José Mellado, África Holguín, María Luisa Navarro, María Isabel González-Tomé, José Tomás Ramos, on behalf of the Madrid Cohort of HIV-Infected Mother-Infant Pairs

**Affiliations:** 1 Paediatrics Department, Hospital Universitario de Getafe, Madrid, Spain; 2 Infectious Diseases Unit, Paediatrics Department, Hospital General Universitario Gregorio Marañón, Madrid, Spain; 3 HIV-1 Molecular Epidemiology Laboratory, Microbiology and Parasitology Department, Hospital Ramón y Cajal-IRYCIS and CIBER-ESP, Madrid, Spain; 4 Paediatrics Department, Hospital Clínico Universitario San Carlos, Madrid, Spain; 5 Obstetrics and Ginecology Department, Hospital Universitario 12 de Octubre, Madrid, Spain; 6 Tropical and Infectious Diseases Unit, Paediatrics Department, Hospital Universitario La Paz, Madrid, Spain; 7 Infectious Diseases Unit, Paediatrics Department, Hospital Universitario 12 de Octubre, Madrid, Spain; Centers for Disease Control and Prevention, UNITED STATES

## Abstract

**Background:**

An increasing number of perinatally HIV-infected women (PHIV) are reaching adulthood and becoming pregnant. Most PHIV women have been exposed to a high number of antiretroviral regimens, and they may have difficulties to achieve viral suppression. Psychosocial problems are not uncommon and could be an important barrier for treatment adherence. The effects of chronic HIV infection and long-term exposure to antiretroviral treatment of PHIV women cause concerns on the developing fetus. The aims of this study were to describe the prevention of mother-to-child transmission strategies in PHIV women and the infant outcomes in the Madrid Cohort of HIV-infected mother-infant pairs.

**Methods:**

All PHIV pregnant women registered in the Cohort that gave birth from 2000 to 2015 were included in the study.

**Results:**

Twenty-eight pregnancies in twenty-two perinatally infected women were registered. Most women were Caucasian and heavily treatment-experienced. Nine cases (32.1%) were at high risk of HIV mother-to-child transmission. Maternal HIV-1 viral load was detectable close to delivery in four women (14.3%). The management of these cases was described, and the treatment strategies were discussed. None of the newborns acquired HIV infection. Eight infants (28.6%) were small for gestational age.

**Conclusions:**

This study included a large series of pregnancies among PHIV women attended according to a youth-centered care model. The challenges in the management of this population by health-care providers were described. Specific strategies to minimize perinatal transmission risks should be addressed in future collaborative studies.

## Background

In Western European countries the efforts in the prevention of mother-to-child transmission of HIV (PMTCT) have led to an important reduction in the rates of new infections in children. [1–4] Cases of infection in children still occur, even if women were receiving antiretroviral treatment (ART) before conception, mainly due to detectable HIV plasma viral load as a consequence of drug resistance or poor maternal adherence to treatment. [5,6]

An increasing number of perinatally HIV-infected women (PHIV) are reaching adulthood and becoming pregnant. Most PHIV women have been exposed to a high number of antiretroviral regimens during pediatric care, and when they become pregnant, they may have difficulties to achieve viral suppression close to delivery. [7–10] Psychosocial problems are not uncommon in this population and could be an important barrier for treatment adherence. [11] The effects of chronic HIV infection and the long-term exposure to ART of PHIV women cause concerns on the developing fetus. [12,13] Moreover, little is known about the risk of HIV-MTCT in PHIV women and the specific clinical management of this population.

The aims of this study were to describe the PMTCT strategies in PHIV women and the infant outcomes in the Madrid Cohort of HIV-infected mother-infant pairs.

## Methods

The Madrid Cohort of HIV-infected mother-infant pairs is a multicenter, prospective and observational study of HIV-1 infected women and their children. Since 2000, mother and infants pairs have been recruited from 8 hospitals in Madrid. The characteristics of the Cohort have been previously described elsewhere. [4]

All PHIV pregnant women registered in the Madrid Cohort who gave birth from 2000 to 2015 were included in the study. Data from PHIV pregnant women since the first visit for pregnancy monitoring until delivery, and data from their infants at birth and during a follow-up period up to 24 months of age were analyzed. Infants were considered HIV-uninfected if they had at least two negative HIV-1 RNA tests, one of them after the age of 3 months. These results were confirmed with at least one HIV-1 antibody assay at the age of 12 to 18 months.

Additionally, the characteristics of PHIV women before pregnancy were requested and obtained from two observational Cohorts: the Madrid Cohort of HIV-infected children and the Spanish Cohort of HIV-infected children and adolescents (CoRISPE). [14,15]

Women were considered to have acquired the HIV infection perinatally if they were diagnosed during childhood in the absence of other risk factors and if their own mothers had been also considered as HIV-infected.

Antiretroviral triple class resistance (TC-DRM) was defined as the extensive drug resistance to the three main antiretroviral families for HIV treatment, where the presence of genotypic resistance mutations was observed for at least one drug from each antiretroviral class [nucleos(t)ide reverse transcriptase inhibitors (NRTIs) non-nucleoside reverse transcriptase inhibitors (NNRTIs) and boosted protease inhibitors (PIs)].

High risk factors for HIV mother-to-child transmission were defined as one or more of the following: (1) a plasma HIV-1 RNA (viral load) higher than 200 copies/mL in two consecutive samples after week 28 of pregnancy (VF after 28 weeks), (2) or any detectable viral load close to delivery, (3) or harbouring TC-DRM. [16]

Newborns were considered small for gestational age (SGA) when birth weight was lower than <10th percentile based on Spanish gender-specific standards. [17] Congenital anomalies at birth were recorded and classified according to EUROCAT (European Surveillance of Congenital Anomalies). [18]

The study was approved by the Institutional Review Board at Hospital Universitario de Getafe, Madrid, Spain.

## Results

### Feature of the study population during pregnancy and delivery

Twenty-eight pregnancies were recorded among twenty-two perinatally-infected women, corresponding to twenty-two first pregnancies and six second pregnancies; all singleton. An integrated management of the pregnant women by the Infectious Diseases, Obstetrics and Paediatrics Department was carried out in all but one case. PHIV women were assisted by psychologist during their visits to the Clinics. The maternal characteristics at conception are described in Table 1. Most women were Caucasian and were heavily ART-experienced.

**Table 1:** Characteristics of 22 perinatally HIV-1 infected women at conception.

**PHIV women characteristics**	
Ethnicity; n (%)	
Caucasian	21 (95.4)
Sub-Saharan	1 (4.5)
Age at first conception (years); median (range)	20 (18–21)
Previous AIDS diagnosis; n (%)	6 (27.3)
**Characteristics at conception of 28 pregnancies**	
Number of previous ART regimens; n (%)	
1–2	3 (10.7)
3–5	5 (17.9)
≥ 6	20 (71.4)
On ART; n (%)	18 (64.3)
CD4 counts; n (%)	
< 200 cells/mm3	3 (10.7)
200–499 cells/mm3	7 (25.0)
> 500 cells/mm3	17 (60.7)
Unknown	1 (3.6)
Viral load (copies/mL); n (%)	
≤ 50	14 (50.0)
51–999	4 (14.3)
1000–9999	2 (7.1)
≥ 10000	8 (28.6)

PHIV, perinatally infected women with HIV; n, number; AIDS, acquired immunodeficiency syndrome; ART, antiretroviral treatment; CD4, lymphocyte T CD4; c/ml, HIV-1 RNA copies per milliliter of plasma.

Nine women (32.1%) were not on ART at the time of conception but it was initiated during pregnancy in eight of these cases. Three of these women had a CD4 count below 200 cells/mm^3^.

The mode of delivery and the pregnancy outcomes are shown in Table 2. There were eight cases of SGA (28.6%). All but one patient were born at term (S1 Table). No birth defects were observed. No cases of perinatal HIV transmission were recorded during the children follow-up.

**Table 2:** Delivery characteristics and outcomes of 28 pregnancies outcomes from 22 perinatally HIV-1 infected women.

**Delivery characteristics**	
Number of deliveries	28
Mode of delivery:	
Vaginal; n (%)	15 (53.6)
Elective Caesaraen section; n (%)	11 (39.3)
Acute Caesarean section; n (%)	2 (7.1)
ZDV intrapartum; n (%)	24 (85.7)
**Pregnancy outcomes**	
Newborn sex male; n (%)	18 (64.3)
Gestational age, median weeks (IQR)	38 (37–40)
Gestational age at birth < 37 weeks; n (%)	1 (3.6)
Birth weight (g); median (IQR)	2920 (2585–3142)
Birth weight; n (%):	
< 2500 g	5 (17.9)
< 1500 g	0
Small for gestational age (%)	8 (28.6)
Breastfeeding; n (%)	0
Postnatal child prophylaxis; n (%):	
Monotherapy	22 (78.6)
Dual or triple therapy	6 (21.4)
None	0
MTCT rate; n (%)	0

IQR, interquartile range; MTCT, mother-to-child transmission; ZDV, zidovudine.

### Detectable VL and presence of resistant viruses during pregnancy

There were 9 cases of high risk for HIV mother-to-child transmission, TC-DRM were documented in three women. In all TC-DRM cases a salvage regimen led to undetectable viral load close to delivery. The characteristics of these cases are summarized in Table 3.

**Table 3:** Description of high risk cases for HIV mother-to-child transmission.

Age at delivery (years)	Year of delivery	ART at conception	Number of previous ART regimens	Previous-to-start/switch HIV-1 RNA VL (copies/ml)	TC-DRM	ART during pregnancy	CD4 count at delivery (cells/mm^3^)	GA at delivery	HIV-1 RNA VL at delivery (copies/mL)	Mode of delivery	Infant prophylaxis
**22**	2007	ABC+ TDF+ ATV/r	7	<50	No	Stop ARV on week 5+5. Start ABC + 3TC + TDF + LPV/r on week 16 +1.	899	38+1	413	VD	ZDV + 3TC + NVP
**20**	2009	No treatment	10	51284	No	Start with ZDV + 3TC + LPV/r on week 12. Switch to ZDV + 3TC + ATV/r + RAL + T20 on week 29+6 because of VF.	561	37+2	<20	EMCS	ZDV
**19**	2010	No treatment	6	5700	Yes	Start with RAL + DRV/r + T20 on week 16 +5.	664	38	<20	ELCS	ZDV
**21**	2011	No treatment	6	Unknown	No	No treatment	Unknown	38+0	44977	EMCS	ZDV + 3TC + NVP
**16**	2012	ABC+ ETV + DRV/r	7	36700	Yes	ABC + ETV + DRV/r	377	36+6	<50	ELCS	ZDV + 3TC + NVP*
**21**	2013	FTC+ TDF + ATV/r	8	435	No	Switch to FTC + TDF + ATV/r + RAL + MRV on week 37+1 because of VF.	307	39+4	<50	ELCS	ZDV + 3TC + NVP
**22**	2013	No treatment	5	32240	No	Start with TDF + FTC + LPV/r on week 4. Switch to TDF + FTC + DRV/r + RAL + MVC on week 23 +4 because of VF.	65	37+4	24300	ELCS	ZDV + 3TC + NVP
**18**	2014	FTC + TDF + LPV/r	7	24709	Yes	Switch to FTC + TDF + ETV + DRV/r + RAL on Week 16 +6.	563	38+2	<20	ELCS	ZDV
**25**	2014	No treatment	14	183200	No	Start with 3TC + ABC + DRV/r on week 7. Switch to 3TC + ABC + DRV/r + RAL on week 28+2 because of VF.	130	37+2	657	ELCS	ZDV + 3TC + NVP

* When maternal viral load close to delivery was known and resulted undetectable, the neonate was switched from the 3-drug combination prophylaxis to a 4-week ZDV regimen.

VL, viral load; c/ml, HIV-1 RNA copies/ml; TC-DRM, presence of drug resistance mutations to the three main ARV families (NNRTI, NRTI and PI) in viruses. GA, gestational age; TC-DRM, antiretroviral triple class resistance; ELCS, elective cesarean section; EMCS, emergency cesarean section; VD, vaginal delivery; 3TC, lamivudine; ABC, abacavir; ATV/r, atazanavir/ritonavir; DRV/r, darunavir/ritonavir; ELCS, elective cesarean section; EMCS, emergency cesarean section; ETV, etravirine; FTC, emtricitabine; TDF, tenofovir dixoproxil fumarate; LPV/r, lopinavir/ritonavir; MRV, maraviroc; NVP, nevirapine; RAL, raltegravir; T20, enfuvirtide; VL, viral load; ZDV, zidovudine.

Four out of six PHIV pregnant women under virological failure after 28 weeks of gestation were not on ART before conception. Although these pregnancies were recognized early, HIV viral load was not fully suppressed at week 28 in all cases. There was a patient who rejected antiretroviral treatment and psychosocial support. Among the other patients, viral load was closely monitored and treatment options were discussed. In four of them a new antiretroviral drug was added including raltegravir. Finally, two of them achieved undetectable viral load close to delivery. Overall, maternal viral load was detectable close to delivery in four women (14.3%). In these four cases, elective cesarean section was the mode of delivery and infants received a combination of three drugs as postnatal prophylaxis.

## Discussion

Our study showed that the situation of PHIV women in Madrid who became pregnant was challenging. They were very young at the time of first conception, with treatment adherence problems as previously described for this population, and also heavily ART-exposed. A high percentage of these women had detectable HIV-1 viral load at conception, and one third of them were on a high risk situation for HIV-1 transmission to their newborns. However, reassuringly, most of the women achieved an undetectable HIV-1 viral load close to delivery and there were not cases of HIV-1 transmission to the second generation. Another observation of our study was the high proportion of SGA newborns in our cohort, even though only one child was born prematurely.

An increasing number of PHIV young people are reaching adulthood throughout Europe. [19] HIV-infected youth may face barriers for treatment adherence and retention in care. [20] Psychosocial barriers, but also, stigma, social or family support and socioeconomic status can impact the long-term outcomes of this population. [21] Moreover, the consequences of long-term exposure to antiretroviral drugs, the use of suboptimal therapies during their childhood, and the effects of chronic inflammation and the immune activation are unknown for both the mothers and the exposed newborns.

It is common that perinatally HIV-infected youth started to be sexually active while still under the care of paediatric health services. [22,23] When analysed, perinatally HIV-infected youth have not always had an adequate knowledge about their disease. [23] Unprotected sexual intercourse may be not so uncommon, leading to unintended pregnancies. [23,24] The discussions with HIV-infected adolescents may not focus only on the prevention of sexually transmitted infections but also on family planning. [25] Preconception care and counseling are very important for pregnancy planning but also to ensure that perinatally infected women are on effective ART to avoid HIV transmission to their partners and their future children. [26]

Nine women (33.4%) were not on ART at conception. Previous reports have showed that it is not uncommon that PHIV women were not receiving ART at conception. An analysis of 44 PHIV pregnant women from the United Kingdom and Ireland showed that only 71% of pregnant women were on ART before gestation. [27] Psychosocial barriers and stigma could be the most important factor in treatment adherence for them. [28] Previous studies from the Madrid Cohort also showed that there were significant and complex psychosocial problems in perinatally HIV-infected youth. [23] Psychosocial and emotional support may be a key element to embrace ART by women once pregnancy is recognized and to improve adherence rates. In this study, six out of the nine women who started ART in pregnancy, achieved an undetectable HIV-1 RNA viral load close to delivery.

High risk situations of HIV-1 transmission, defined as TC-DRM, virological failure after week 28 of pregnancy and detectable HIV-1 viral load close to delivery, were also common. For these situations, an individual approach in the selection of ART regimens in combination with psychosocial support could be useful. The use of new antiretroviral drugs as salvage therapies based on the results of the genotypic resistance tests in women with TC-DRM should be considered. Integrase inhibitors may play an important role in achieving a more rapid decline in plasma viral load to undetectable levels. However, there were four cases of pregnant women with non-suppressed HIV viral load close to delivery. Strategies to optimize adherence, including the consideration of the women preferences, are crucial and may be challenging in high risk situations. Elective Caesarean section and combined neonatal prophylaxis could also be very important measures to avoid HIV-1 transmission. [29]

Special attention has been focused on models that integrate obstetrics and mental health services into the HIV care setting for meeting the needs of PHIV pregnant women. [30] This series also showed the results of a multidisciplinary approach for these PHIV pregnant women, with includes the coordinated action of HIV, obstetrics and mental health care providers previously experienced in HIV-infected youth. The team tried to give an integrated response to the special needs and concerns of each woman and could be useful for reaching better outcomes. However, this observational study could not evaluate the real impact of this youth-centered model in the outcomes of PHIV pregnant women.

In previous studies there were not important gender disparities in HIV-exposed infants, [3,4] although it has been published that the risk of acquisition of HIV may be higher for female infants than for male ones. [31,32] A high rate of male infants (64%) born from PHIV women has been observed in this study. However, the interpretation of this result requires considerations. Firstly, the number of PHIV women and infants included were small, and secondly, the rates of miscarriage or termination of pregnancies were not analyzed in this study. To our knowledge, previous studies on PHIV pregnant women have not well assessed this topic. The gender of these infants may be further evaluated in PHIV women studies.

Prematurity and fetal growth restrictions leading to low birth weight have been associated with an overall increase of neonatal and long-term morbidity and mortality in neonates. [33,34] The rates of SGA among neonates from PHIV women in this study (28.6%) were higher than expected when compared to non-HIV-infected young women in Spain. [35] In a previous report from US, the SGA risk was higher in PHIV women compared to behaviorally acquired HIV-infected women [adjusted OR of 5.7 (95% CI = 1.03–31.61)]. [12] Although the numbers are small to draw conclusions, it may be a matter of concern if confirmed in other studies with larger number of PHIV women. Further studies are needed to determine the causal mechanisms and the possible role of long-term exposure to ART [36] and chronic inflammation. [37] HIV-exposed children born SGA may thus warrant an appropriate follow-up throughout adulthood.

There are several limitations to our study. The number of pregnancies reported makes our findings entirely descriptive. In this study we were unable to address whether the pregnancies were planned or not, and how this could affect the outcomes. We have neither analyzed the rate or causes of previous miscarriages or stillbirths. The use of illicit drugs or tobacco during pregnancy was not assessed. We did not either specifically assess obstetric complications such as gestational diabetes or comorbidities such as sexually transmitted infections.

In summary, this study provides a large series of pregnancies in PHIV women, reflecting some challenges and programmatic implications of this unique population for health-care providers. The characteristics and outcomes of these PHIV women attended according to a youth-centered care model were described. Additionally to this model, specific strategies should be addressed in future or collaborative studies in order to improve the management of high-risk patients for HIV-1 transmission and thus minimize the vertical transmission rate.

## Supporting information

S1 Table Baseline characteristics of HIV-exposed newborns. (DOCX)
