# Supplementary material for: Pregnancy outcomes in perinatally HIV-infected young women in Madrid, Spain: 2000-2015
Source: PLoS One. 2017 Aug 25;12(8):e0183558. doi: 10.1371/journal.pone.0183558 (PMC5571961; doi:10.1371/journal.pone.0183558)
Supplement: S1 Table — (DOCX) [file pone.0183558.s001.docx]

**S1 Table. Baseline characteristists of HIV-exposed newborns.**

| **Case Number** | **Gestational age (weeks + days)** | **Gender** | **Newborn Weight (grames)** | **Weight Percentile** |
| --- | --- | --- | --- | --- |
| 1 | 38+1 | M | 2930 | 28 |
| 2 | 40+1 | F | 3310 | 51 |
| 3 | 40+6 | M | 3220 | 27 |
| 4 | 40+2 | F | 2850 | 12 |
| 5 | 39+2 | F | 2400 | 2 |
| 6 | 38+5 | F | 2600 | 8 |
| 7 | 41+3 | M | 2540 | 1 |
| 8 | 39+4 | M | 3000 | 19 |
| 9 | 38+0 | M | 3250 | 59 |
| 10 | 37+4 | M | 2320 | 6 |
| 11 | 37+0 | M | 2820 | 42 |
| 12 | 37+0 | M | 2500 | 19 |
| 13 | 40+0 | F | 3180 | 39 |
| 14 | 37+2 | F | 2900 | 54 |
| 15 | 36+6 | M | 2880 | 54 |
| 16 | 38+1 | F | 2380 | 5 |
| 17 | 37+0 | M | 2850 | 45 |
| 18 | 38+0 | M | 2300 | 2 |
| 19 | 38+6 | M | 2910 | 18 |
| 20 | 40+0 | F | 3590 | 80 |
| 21 | 37+0 | M | 3120 | 69 |
| 22 | 39+4 | F | 3000 | 27 |
| 23 | 37+0 | F | 3410 | 92 |
| 24 | 37+2 | M | 2160 | 3 |
| 25 | 40+1 | M | 3810 | 84 |
| 26 | 38+4 | M | 2990 | 28 |
| 27 | 39+0 | M | 3130 | 33 |
| 28 | 40+6 | M | 2930 | 9 |

M, male; F, female.
